# Transient Receptor Potential Vanilloid Subtype 1: Potential Role in Infection, Susceptibility, Symptoms and Treatment of COVID-19

**DOI:** 10.3389/fmed.2021.753819

**Published:** 2021-11-04

**Authors:** Filippo Liviero, Manuela Campisi, Paola Mason, Sofia Pavanello

**Affiliations:** Occupational Medicine, Department of Cardiac, Thoracic, Vascular Sciences and Public Health, University Hospital of Padua, Padova, Italy

**Keywords:** TRPV-1, SARS-CoV-2, COVID-19, SNPs, pollution, inflammation, therapy

## Abstract

The battle against the new coronavirus that continues to kill millions of people will be still long. Novel strategies are demanded to control infection, mitigate symptoms and treatment of COVID-19. This is even more imperative given the long sequels that the disease has on the health of the infected. The discovery that S protein includes two ankyrin binding motifs (S-ARBMs) and that the transient receptor potential vanilloid subtype 1 (TRPV-1) cation channels contain these ankyrin repeat domains (TRPs-ARDs) suggest that TRPV-1, the most studied member of the TRPV channel family, can play a role in binding SARS-CoV-2. This hypothesis is strengthened by studies showing that other respiratory viruses bind the TRPV-1 on sensory nerves and epithelial cells in the airways. Furthermore, the pathophysiology in COVID-19 patients is similar to the effects generated by TRPV-1 stimulation. Lastly, treatment with agonists that down-regulate or inactivate TRPV-1 can have a beneficial action on impaired lung functions and clearance of infection. In this review, we explore the role of the TRPV-1 channel in the infection, susceptibility, pathogenesis, and treatment of COVID-19, with the aim of looking at novel strategies to control infection and mitigate symptoms, and trying to translate this knowledge into new preventive and therapeutic interventions.

## Introduction

COVID-19, a new human respiratory disease that continues to kill millions of people, is a worldwide public health challenge. Its infectious agent, SARS-CoV-2, diverges from other coronaviruses in some structural characteristics that render this virus more pathogenic and transmissible. Of the four structural proteins, the spike protein (S) plays the fundamental role in cell receptor recognition and subsequent entry of the virus. The discovery that S protein encompasses two ankyrin binding motifs (S-ARBMs) and some transient receptor potential (TRP) cation channels present the same ankyrin repeat domains (TRPs-ARDs) (1), it may be postulated that the transient receptor potential vanilloid subtype 1 (TRPV-1), the most studied member of the TRPV channel family, can play a role in binding SARS-CoV-2. This hypothesis is strengthened by studies revealing that other respiratory viruses bind the TRPV-1 on sensory nerves and epithelial cells in the airways (2). Furthermore, the pathophysiology in COVID-19 patients is similar to the effects generated by TRPV-1 stimulation (3). Finally, treatment with agonists that down-regulate or inactivate TRPV-1 may have a beneficial effect on impaired lung function (3–5), and clearance of infection (6). In this review, we explore the role of TRPV-1 channel in the infection, susceptibility, pathogenesis, and treatment of SARS-CoV-2 infection.

## TRPV-1

TRPV-1 is a nonselective cationic ligand-gated channel with high permeability to Ca^2+^, extensively expressed on neuronal and non-neuronal cell membranes, including immune cells and type C sensory nerve fibers of air route (upper and lower lung tract and parenchyma), where they act as molecular sensors to differentiate temperature, noxious substances, and pain. This was a revolutionary discovery which earned David Julius the victory of the 2021 Physiology/Medicine Nobel Prize. TRPV-1 participates (through the generation of Ca^2+^ dependent signals) in mechanisms that contribute to the defense of the airways such as cough and mucociliary clearance (7, 8). The activation of TRPV-1 mainly allows extracellular Ca^2+^ entrances into neuronal cells, with release of neurotransmitters, the excitability of the membrane and contraction of airway smooth muscle (9). It is also considered a “pathological receptor” that plays an important role in the transduction of noxious stimuli and in the maintenance of inflammatory conditions (10). In fact, TRPV-1 is involved in various inflammatory conditions, such as in inflammatory bowel disease (IBD), cutaneous neurogenic inflammation, brain inflammation, allergic asthma, colitis, arthritis, hypersensitivity, chronic obstructive pulmonary disease (COPD), and autoimmune diseases (11).

TRPV-1 works as a multisensory receptor for damage signals and following exposure to inhaled particles, such as allergens, cigarette smoke, air pollutants and virus too. Inflammation of the airways is supported by the transfer of the signal from neuronal fibers TRPV-1-positive to immune cells (12, 13). TRPV-1 can also be triggered by exogenous mediators such as capsaicin (CPS), resiniferatoxin, temperature (higher than 40°C), acidic conditions (e.g., citric acid), and endogenous mediators, including bioactive lipids, mainly produced during inflammation (e.g., prostaglandins E2 (PGE2), thromboxanes, and leukotrienes, three classes of arachidonic acid derivatives). Furthermore, activation of TRPV-1 boosts the release of various pro-inflammatory molecules, including neuropeptides substance P (sP) and cytokines such as interleukin 6 (IL-6), the same involved in the pathophysiological events affecting the COVID-19. All the above hints envisage the involvement of TRPV-1 in COVID-19 infection (3).

## TRPV-1 in Viral Infections

TRPV-1 expression is significantly activated by several viral infections, including those through the respiratory route, i.e., human respiratory rhinovirus (HRV) and syncytial virus (RSV), or even through other routes i.e., measles virus (MV), hepatitis C virus (HCV), herpes simplex virus type 2 (HSV-2), herpes simplex virus type 1 (HSV-1), and varicella-zoster virus (VZV) (14, 15). This therefore suggested that TRPV-1 plays a central role in host-pathogen contacts including the binding, entry and replication of the virus. Recently, the involvement of TRPV-1 during Chikungunya virus (CHIKV) infection was studied in host macrophages (16).

**Figure 1:**
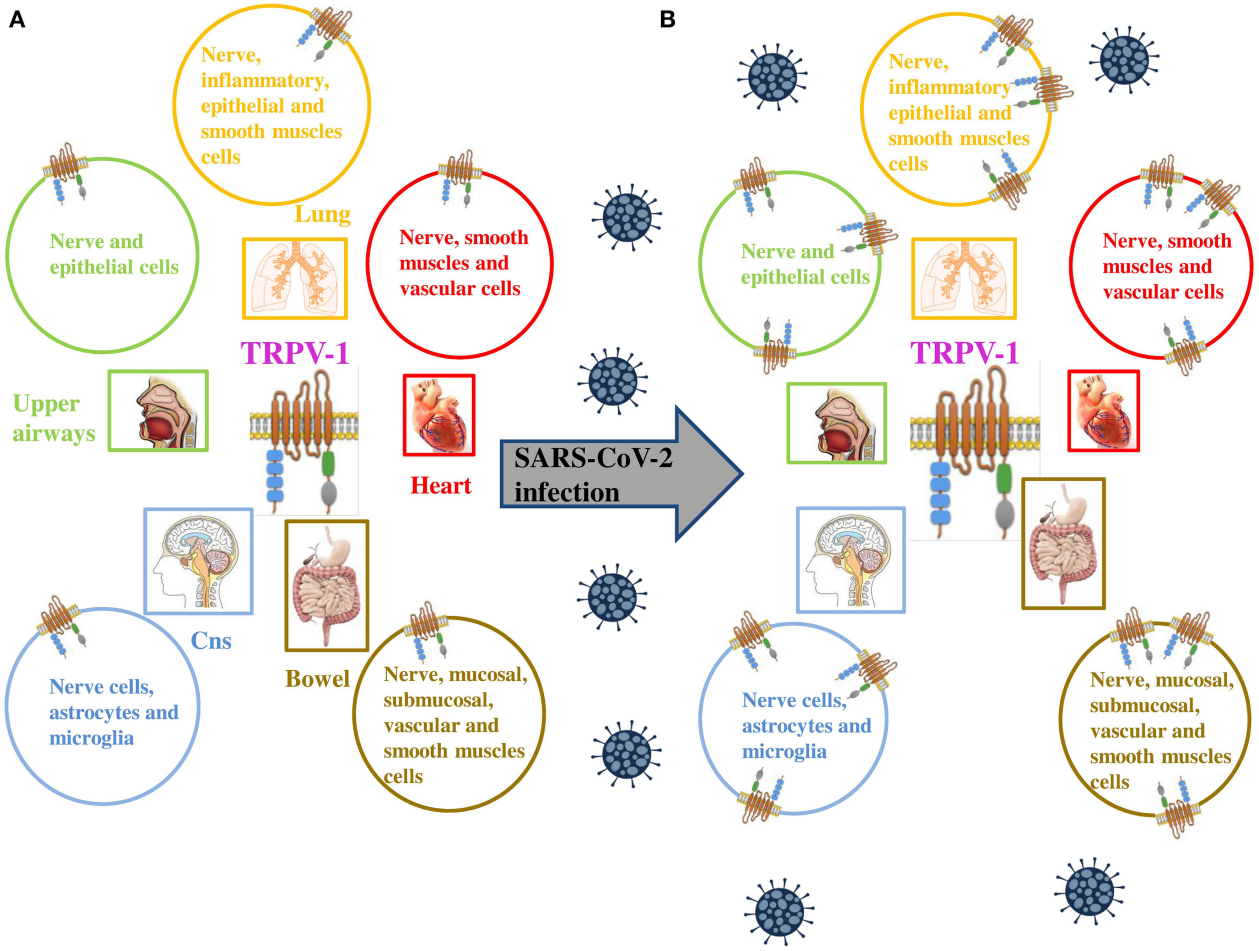
**(A)** TRPV-1 is extensively expressed on neuronal and non neuronal cell membranes, including immune cells and type C sensory nerve fibers of the airways (upper and lower respiratory tract and lung parenchyma). **(B)** TRPV-1 has been found to be significantly upregulated in numerous viral infections, similarly SARS-CoV-2 is proposed to upregulate the expression of the channel on neuronal and non neuronal cell membranes of infected patients. Adapted from (9).

Furthermore, likewise COVID-19, CHIKV is a single-stranded RNA virus, and generates symptoms, fever, including high fever, nausea, vomiting, headache, rashes, polyarthralgia, and myalgia (17–19), comparable to that of COVID-19. Results showed that TRPV-1 was upregulated by CHIKV infection. The involvement of TRPV-1 in CHIKV was confirmed by using specific modulators the 5'-iodoresiniferatoxin (5'-IRTX, a TRPV-1 antagonist) and resiniferatoxin (RTX, a TRPV-1 agonist). The results indicate that TRPV-1 inhibition leads to a reduction in CHIKV infection, whereas TRPV-1 activation significantly enhances CHIKV infection (16). Furthermore, Sanjai Kumar and co-workers demonstrated that CHIKV infection regulated Ca^2+^ influx through TRPV-1 resulting in a higher production of pro-inflammatory TNF and IL-6 the same during COVID-19 infection. These findings, therefore, suggest the involvement of TRPV-1 in other viral infections, including COVID-19 (Figure 1).

## Inflammation in SARS-COV-2 Infection and Potential Role of TRPV-1

SARS-CoV-2 induces an alveolar-interstitial inflammation with a high risk of acute pulmonary edema or acute respiratory distress syndrome. The clinical signs of COVID-19 are consistent with those observed in viral pneumonia (20). These pulmonary changes are likely responsible for both systemic and localized immune responses leading to a hyperinflammatory state. The mortality rate in patients with SARS-CoV-2 infections is related to virally driven “cytokine storm” that results from a severe immune reaction in the lungs as measured by high levels of inflammatory markers (c-reactive protein, serum ferritin) and cytokine levels (IL-6, IL-2, IL-7, IL-10, GSCF, IP10, MCP1, MIP1A, and TNFα) in the plasma (21). Underlying physiological events leading to mortality have been hypothesized to be closely linked to the TRPV-1 expressing neuronal system in the lungs. The respiratory tract (higher and lower) is densely populated by sensory afferents originating from neurons in the nodose (vagal) ganglia (VG) and dorsal root ganglia (DRG). Many of the neurons in these ganglia express high levels of the TRPV-1 ion channel. The crosstalk between TRPV-1 positive nerve fibers and immune cells is critical in mediating inflammation of the airways following exposure to either inhaled allergens or viral infection (12, 22). A recent study has demonstrated that respiratory viral infections (by rhinovirus, respiratory syncytial virus or measles virus) can upregulate TRPV-1 receptors by channel specific mechanisms (2). This upregulation can drive an inflammatory cascade in the lungs leading to airways hyperactivity and is dependent on the viral load and duration of infection. Interestingly, treatment with TRPV-1 antagonist in this study significantly inhibited TRPV-1 upregulation post viral infection. The interaction of SARS-CoV-2 virus with TRPV-1 receptors has not yet been investigated but given the respiratory pathophysiology in COVID-19 cases, may exhibit similar mechanisms that can result in sensitizing TRPV-1 receptors resulting in hyper-inflamed lungs and associated complications. Indeed activation of TRPV-1 enhances the release of several pro-inflammatory molecules, including sP, and cytokines such as IL-6. Moreover, pro-inflammatory substances have reported to be upregulated in COVID-19 cases and reflect the severity of the disease (23).

## Susceptibility to COVID-19 Infection

### Pollution

Two big studies conducted in France examined the incidence of myocardial infarction (MI) admission during the COVID-19 pandemic, in particular the periods before and after the lockdown in France (24, 25). The first study conducted in 22 centers in France identified a significant decline of admission for MI (including ST-segment and non-ST segment raise MI) during COVID-19 national lockdown. Both studies reported 30% (24) and 20% (25) drop of MI, the latter observed in two region of France (“Hauts-de-France” and “Pays de-la-Loire”). The authors concluded that the reduction in hospital admissions was influenced by the decrease in air pollution, a well-known trigger of acute MI (26).

Numerous epidemiological studies have consistently highlighted associations between mortality and morbidity due to cardiopulmonary diseases and increased air pollutants (27, 28). These relationships, which are more reliable for particulate matter (PM) and are often observed within hours of PM concentration peaks in urban air, suggest that very fast events should take place (29, 30). A number of authors have suggested neurological mechanisms to explain such short-term toxicity of PM (27, 31–36) with TRPV-1 localized on vagal bronchopulmonary C-fibers endings in the lung, as primarily responsible for eliciting centrally mediated reflexes (37).

**Figure 2:**
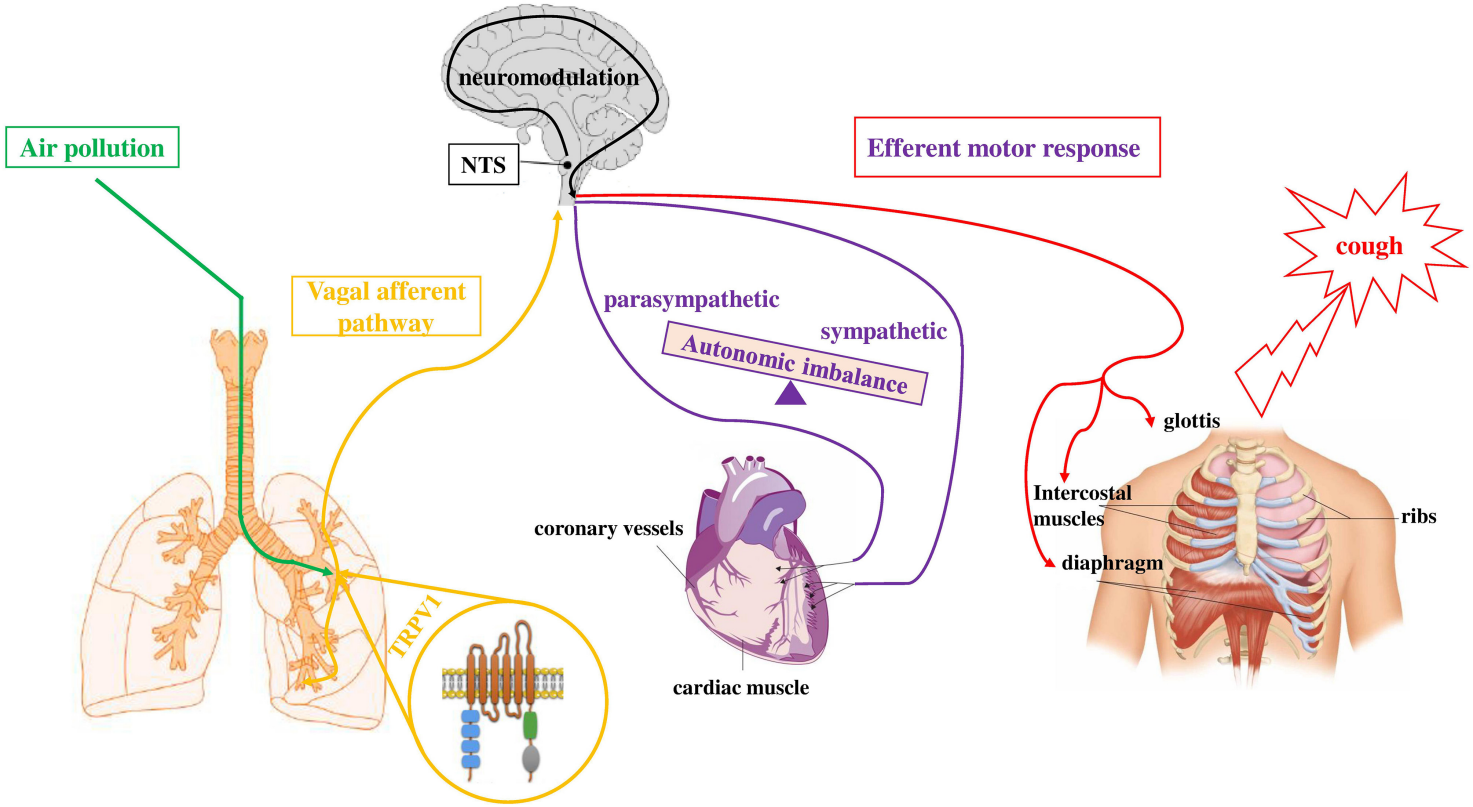
Simplified depiction of how exposure to air pollutants is proposed to sensitize the sensory to autonomic reflex arc and alter subsequent responses. Inhaled component of air pollution (i.e., DEP), directly sensitizes TRPV-1 (green pathway) located on vagal bronchopulmonary C-fibers endings. Activation of airways sensory nerves (yellow pathway) stimulates neurons in the midbrain (NTS) and, through a neuromodulation process in the CNS, causes the efferent motor responses (red and violet pathways) to the heart (autonomic imbalance) and to respiratory muscles (cough). Adapted from (141).

*In vitro* and *in vivo* studies showed that TRP channels are activated by air contaminants. We recently demonstrated that air pollutants, such as DEP, directly interact with TRPV-1 and cause channel opening (38). Furthermore, the inhalation of environmental (39) and diesel exhaust particulate (DEP) (36) stimulate TRPV-1 causing changes in cardiac rhythm, electrocardiogram (ECG) morphology, and decreased heart rate variability (HRV). These results may be explained considering an imbalance of autonomic heart control (in favor of sympathetic activity), with centrally-mediated reflexes, *via* the afferent unmyelinated C-fibers, which are in turn activated by PM. In line with this hypothesis, a reduced HRV was observed in susceptible individuals after short-term exposures to PM (40). Furthermore, in patients taking ß-blockers, which regulated the sympathetic activity, HRV reduction by PM exposure was not detected (41). Our recent data (38) indicate that signals from airways sensory nerves (i.e., DEP which directly activate TRPV-1 and also endogenous mediators such as prostaglandin E2 (PGE2) and bradykinin (BK) which are considered to be indirect sensitizers of the channel), when they joined the central nervous system (CNS) can affect the autonomic impulse to the heart (Figure 2). All this evidence postulates a proof of concept that explains the indication that peaks of pollutants are associated with short-term cardiovascular adverse events in susceptible subjects, as for example COVID-19 patients.

### Interconnection Between ACE2, TMPRSS2 and TRPV-1

**Figure 3:**
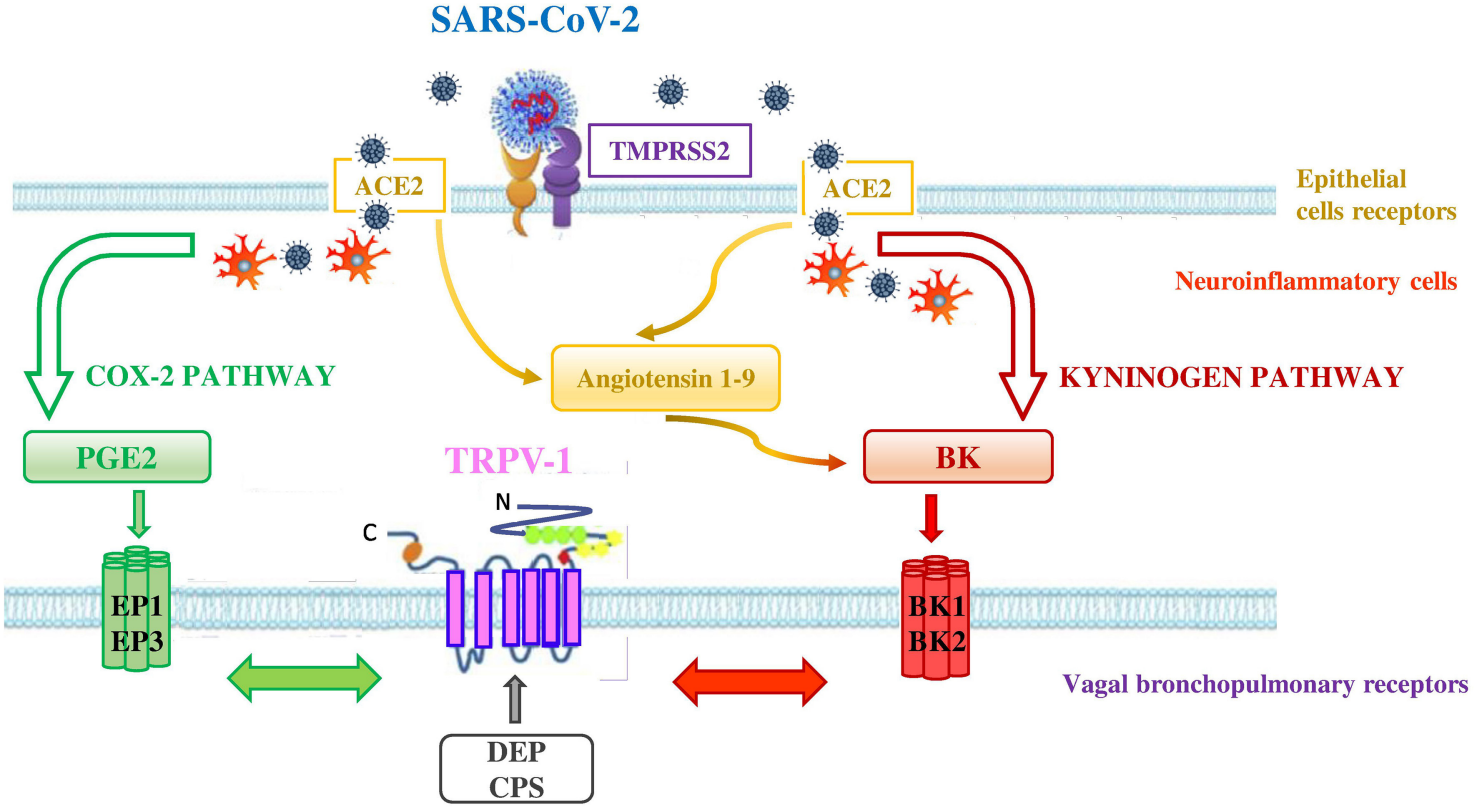
Interconnection between ACE2, TMPRSS2, and TRPV-1 in SARS-CoV-2 infection. SARS-CoV-2 uses the ACE2 receptor for entry into lung epithelial cells and the host cell serine protease TMPRSS2 for priming the S protein. ACE2 and TMPRSS2 may interact with TRPV-1 through the activation of COX-2 and kininogen pathways. - COX-2 pathway (green pathway). TRPV-1 sensitization is due to the interaction between TRPV-1 and EP receptors (i.e., EP1 and EP3) stimulated by the increase of PGE2 levels in the lungs of affected COVID-19 patients. SARS-CoV-2 interaction with neuroinflammatory cells increases levels of PGE2 a potent inflammatory mediator that is generated by COX-2 conversion of arachidonic acid.- kininogen pathway (red pathway). TRPV-1 sensitization is due to the interaction between TRPV-1 and BK receptors (i.e,. BKB1 and BKB2) stimulated by the increase of BK levels in the lungs. SARS-CoV-2 interaction with neuroinflammatory cells increases levels of BK, referred to as a “Bradykinin Storm.” BK is produced from an inactive pre-protein kininogen through activation by the serine protease kallikrein. The upregulation of ACE2 in patients with severe symptoms of COVID-19 increases Angiotensin 1–9 levels that in turn raise the levels of BK. High levels of PM in air pollution, such as DEP, directly interact with TRPV-1 (gray arrow) by modulating its activity and increasing its sensitization. This interaction could worsen the outcome of COVID-19 disease in affected patients. Adapted from (42).

That TRPV-1 interacts with other receptors is not new (42). TRPV-1 may interact with Angiotensin-converting enzyme 2 (ACE2) and transmembrane protease-serine 2 (TMPRSS2) through the activation of cyclooxygenase 2 (COX-2) and kininogen pathways (Figure 3). ACE2 and TMPRSS2 are broadly documented as key cellular receptors of SARS-CoV-2 to conquer target cells (43). In particular, SARS-CoV-2 spike protein is processed by TMPRSS2 which favors its binding to ACE2, expressed on epithelial lung cells (44).

In the COX-2 pathway, TRPV-1 sensitization may be achieved when SARS-CoV-2, by interacting with neuroinflammatory cells, increases levels of PGE2, a potent inflammatory mediator that is generated by the effect of COX-2 on arachidonic acid. High PGE2 levels lead to prostaglandin receptors 1 (EP1) and 3 (EP3) stimulation and subsequent TRPV-1 sensitization. The EP1 and EP3 are regarded as stimulatory receptors as their activation leads to stimulation of the cell concerned, such as contraction in the smooth muscle cell or activation of the neuron. ACE2 that is a negative regulator of the classical angiotensin-converting enzyme (ACE) in the renin-angiotensin system (RAS) was discovered to be dysregulated (decreased levels of ACE and increased levels of ACE2 in the lung cells) in patients presenting severe symptoms of COVID-19 (45). In addition, a significant increase of bioactive lipid levels modulating lung inflammation of severe COVID-19 patients, compared to healthy controls, has been reported (46). The Authors highlighted in COVID-19 patients, a predominance of cyclooxygenase metabolites, in particular significant levels of PGE2, and also increased levels of leukotrienes, compared to controls (46). These products of inflammation are able to activate TRPV-1.

In the kininogen pathway SARS-CoV-2, by interacting with neuroinflammatory cells, increases levels of BK, which is produced from an inactive pre-protein kininogen through activation by the serine protease kallikrein. High BK levels lead to BK receptors stimulation and subsequent TRPV-1 sensitization on bronchopulmonary C-fibers. There are two types of receptors for BK in the body, the BKB1 receptor which is inducible and is expressed by the presence of inflammation and tissue damage (47), and the BKB2 receptor which is present constitutively (48). Both BKB1 and BKB2 receptors exert their effect by coupling to G proteins and activating phospholipase C or A2. The activation of phospholipase C leads to the sensitization of TRPV-1 through protein kinase C (49, 50). Furthermore, the upregulation of ACE2, in patients with severe symptoms of COVID-19 (45), increases Angiotensin 1–9 levels that in turn rise the levels of BK in the cells (referred to as a “Bradykinin Storm”), comporting a dysregulated BK signaling in COVID-19 patients (51) with further TRPV-1 sensitization.

Furthermore, we demonstrated that the air pollutant, DEP, directly interacts with TRPV-1 contributing to channel opening (38). Therefore, inhalation exposures to high levels of pollution during SARS-CoV-2 infection could worsen the outcome of COVID-19 in affected patients, directly modulating the activity of TRPV-1.

### Susceptibility of Elder People to SARS-CoV-2

Studies on knockout (TRPV-1–/–) mice or using a pharmacological block with TRPV-1 antagonist (capsazepine) or agonist such (resiniferatoxin) have revealed that TRPV-1 presents an anti-inflammatory function and a decreased systemic inflammatory response, by reducing the production of TNFα, on a systemic inflammatory animal model on which a “cytokine storm” was induced. The anti-inflammatory activity gave however way to a pro-inflammatory activity in elderly rats. In particular, TRPV-1 expression was found to be upregulated in the lungs of rats, in relation to not only the progress of pathology but also with age, revealing a primarily anti-inflammatory role of TRPV-1 in young and a pro-inflammatory function in the elderly (52).

The pro-inflammatory role of TRPV-1 in the elderly might contribute to aggravate the incidence of COVID-19 fatality associated with older patients, especially people over 65-years-old. This, along with an overall deterioration of immune function and the higher rate of comorbidity, making the elderly more susceptive to infections, could help to clarify the progression and unbalanced impact of COVID-19 in the elderly.

## Symptoms

The most common symptoms of COVID-19 are fever, cough, dyspnea, altered sense of taste/smell, palpitations. Less common symptoms include: myalgia and arthralgia, fatigue, rhinorrhea/nasal congestion, chest tightness, chest pain and hemoptysis, gastrointestinal symptoms, sore throat, headache, dizziness, neurological symptoms, ocular symptoms, audio-vestibular symptoms, cutaneous symptoms (53). While in severe cases patients with COVID-19 at admission in the hospital the most common symptoms are fever, cough, and/or shortness of breath, in mild or moderate disease are headache, loss of smell, nasal obstruction with cough. Overall, the prevalence of symptoms was highest in people aged 30–60 years; the most common atypical presentation in older adults was confusion. Most of these symptoms are associated with pathways controlled by TRPV-1.

### Cough

Cough is the major COVID-19 symptom (54), not necessarily associated with severity. The cough reflex is initiated by activation of TRPV-1 receptors on vagal bronchopulmonary C-fibers endings which are mainly involved in airways reflex responses and primarily responsible for “detecting” inhaled toxicants' presence. In effect, TRPV-1 represents a portal of entry to respiratory tract irritation and reflex responses induced by inhaled oxidant agents (55, 56), particulate air pollution (39), and cigarette smoking (57). Moreover, patients suffering from chronic cough exhibit increased levels of TRPV-1 positive cells in the airways. Interestingly, TRPV-1 upregulation in neuronal cell cultures, infected by rhinoviruses, may explain cases of cough hypersensitivity syndrome following airway viral infections (post-viral vagal neuropathy), regardless of inflammation (58). Prevalence of post-COVID-19 cough varied widely between studies (59–61). However, there's a growing opinion that vagal neuroinflammation caused by the virus is closely connected to the development and persistence of COVID-19 cough (62).

A way to quantification cough and evaluate the effect of pharmacological intervention in cough investigation is the cough challenge (63). Inhalation cough challenge, the most commonly employed method to assess cough reflex sensitivity, implicates the inhalation of tussive agents and the subsequent counting of the induced coughs number (64). CPS mainly acts on TRPV-1, thus the CPS cough challenge has been applied to investigate TRPV-1 function *in vivo* measuring cough response (63). During an upper respiratory infection, a temporary increase in cough sensitivity to inhaled CPS has been demonstrated, moreover CPS sensitivity has been positively associated with the cough severity score (65). Our group recently demonstrated that cough reflex induced by CPS can be modulated by inhalation of endogenous mediators of TRPV-1, PGE2, and BK, in healthy subjects (38). The upregulation and subsequent modulation of TRPV-1 by lung inflammation products, i.e., PGE2, and BK, during and following airways viral infections, including COVID-19, may explain hypersensitivity of the cough reflex during the period of illness and after COVID-19 (post-viral vagal neuropathy).

### Persistent Fatigue

TRPV-1 is involved in persistent fatigue, a common symptom following SARS-CoV-2 infection (66). Particularly interesting is that TRPV-1 ligands, i.e., CPS and n-tert-butylecyclohexanol, are able to alleviate chronic fatigue syndrome's (CFS) symptoms. The inhibition of TRPV-1 channel and the subsequent modulation of pain perception, neuroendocrine function, oxidative stress, and immune function, are believed to be involved in these beneficial effects. N-tert-butylcyclohexanol, an antagonist of the TRPV-1 channel, is more effective in reducing CFS symptoms than CPS (67).

### From Palpitation to Heart Attack

One of the major complications among COVID-19 patients includes cardiac arrhythmias. The commonest arrhythmia is sinus tachycardia which is usually associated with palpitations causing discomfort to patients. One case of COVID-19 with clinical features of autonomic dysfunction in the form of sinus arrhythmia, postural hypotension, intermittent profuse sweating, constipation, erectile dysfunction, and squeezing sensation in the chest, was recently described (68). Another case of a 58-year-old COVID-19 patient with a significant decrease in heart rate and a paradoxical decline in HRV investigated at 24 h ECG monitoring (69) was published. Only one study evaluated the presence of cardiac autonomic dysfunction in hospital COVID-19 patients (70) founding an increased parasympathetic activity in COVID-19 patients compared to healthy controls as demonstrated by the increase in time domain variables of HRV. Unlike the time domain variables, authors found that frequency domains of HRV, specifically the Low Frequency and High Frequency ratio (LF/HF) (traditionally considered a marker of sympathovagal balance in the cardiovascular system), weren't different between the COVID-19 subjects and the healthy subjects. A case of postural tachycardia syndrome was described several months after confirmed SARS-CoV-2 infection (71). Anecdotal cases of autonomic dysfunction (i.e., palpitations, fatigue, dizziness, diarrhea, recurrent presyncope episodes) following viral SARS-CoV-2 infection are emerging (72). Furthermore, a 58% increase of out-of-hospital cardiac arrest in COVID-19 cases out of a total of 9,806 reported in some provinces of Lombardy, the Italian region most affected by SARS-CoV-2 was identified during the first 40 days of the first wave of the outbreak (February 21st through March 31st, 2020), compared with those that occurred during the same period in 2019. The cumulative incidence of out-of-hospital cardiac arrest in 2020 was strongly associated with the cumulative incidence of COVID-19 and the increase in the number of cases of out-of-hospital cardiac arrest followed the time course of the COVID-19 outbreak (73). Another study conducted in Emilia Romagna region (one of the most severely hit areas of Italy), during the first wave of the COVID-19 pandemic observed an increase in the out-of-hospital cardiac mortality (74). Furthermore, a study conducted in Wuhan, China, reports that cardiac damage also occurs in about 20% of COVID-19 hospitalized patients (75).

TRPV-1 is among the TRP channels involved in the activation of airway sensory nerves causing variability in the autonomic efferent pathways that are resolved centrally at the level of the mid-brain. This causes cardiovascular function changes i.e., alterations of cardiac rhythm and of ECG morphology (36, 39). HRV spectral analysis is a valuable tool for the assessment of cardiovascular autonomic function and to check out fluctuations in autonomic tone. Changes in cardiac autonomic activity are thought to be a common pathway leading to increased morbidity and mortality from various disorders, including cardiovascular disease. Indeed, data from literature sustain the assumption that decreased HRV precedes the evolvement of a number of cardiovascular disease risk factors (76). Plethora of evidence are available in the literature demonstrating autonomic dysfunction in other infectious diseases (77–91).

Recently our group (38) identified a mechanism, which is operative *in vivo* in healthy subjects, by which sensitization of airways sensory TRPV-1 channels by inhalation of endogenous mediators of the channel PGE2 and BK dysregulates autonomic cardiac rhythm increasing sympathetic heart activity. We have demonstrated that an increase in sympathetic activity can be generated by stimuli that are also able to sensitize airways TRPV-1. This brings a proof of concept that signals from vagal bronchopulmonary C-fibers, once they are integrated at the CNS level, can modify autonomic drive to the heart, as was previously demonstrated in animal models.

Therefore, the increase in cardiac arrest that emerged during the COVID-19 pandemic, could be closely related to a potential autonomic dysfunction of cardiac rhythm regulation, caused by TRPV-1 sensitization.

### Gastrointestinal Symptoms

#### Smell and Taste Disorders

Smell and taste disorders are very common in COVID-19 (92–96). The nasal cavity expresss high levels of TRPV-1 trigeminal receptors so that the intranasal trigeminal system is considered the third chemical sense with olfaction and gustation (97). TRPV-1 is among the TRP channels mainly involved in the transduction of noxious sensation and is activated by pungent odorous substances (97). TRP channels are also involved in the process of gustation (98). Indeed associations have been observed between TRPV-1 genetic variant rs8065080 (C>T, Val585Ile) polymorphism, the same we analyzed in our previous work (99), in modulation salt taste perception (100). The CPS agonist of TRPV-1 is also implicated in the modulation of smell and taste with sensory (olfactory) and sensitive (trigeminal) perceptions coming together (101). In addition, most aroma compounds have sensitive peculiarities linked to nasal hyper-reactivity to strong aroma (sometimes identified as “hyperosmia” by patients who present sino-nasal inflammation).

Nasal obstruction alone is relatively frequent in COVID-19. In two studies, nasal obstruction was often reported, but not associated with olfactory dysfunction (102, 103). In rhinitis, the nasal itch is related to TRPV-1 (104). Mucus hypersecretion and inflammation are also associated with TRPV-1 sensitization (2, 105). CPS was found to be a choice as therapy for non-allergic rhinitis (106, 107).

#### Anorexia

Loss of appetite is frequent and could be severe in COVID-19 (108). TRPV-1 is also involved in appetite through control of appetite hormone levels or stimulation of gastrointestinal vagal afferent signaling (109).

#### Nausea, Vomiting, and/or Diarrhea

Nausea, vomiting, and/or diarrhea are rather frequent symptoms of COVID-19 (108). TRPV-1 activation leads to nerve fibers' release of substances such as tachykinins that increase gastric motility and stimulate gastric emptying (110). CPS can promote gastroesophageal and abdominal pain, pyrosis, bloating, and/or dyspepsia through TRPV-1 (111–113).

#### Pain

Myalgia, back pain, widespread hyperalgesia, and headache are often concomitant with COVID-19 infection (96, 114). TRPV-1 is implicated in acute and chronic pain and migraine (115, 116).

## Genetic Susceptibility to SARS-CoV-2 Infection and Symptoms

Genetic factors could explain the variability in COVID-19 symptoms. Single nucleotide polymorphisms (SNPs) in the TRPV-1 gene modulate the functional asset of the channel and contribute to different responsiveness to the agonist CPS *in vitro* (56). Our group recently demonstrated that multiple TRPV-1 polymorphisms explain the variability in cough test sensitivity to CPS in healthy subjects (99). In particular, four combined SNPs: I315M (rs222747); I585V (rs8065080); T469I (rs224534); P91S (rs222749) confer the major CPS sensitivity *in vivo*. Then, the presence of a minimum of two polymorphisms, the 91S combined with 315M or with 585I, was sufficient to produce a significant effect at the CPS concentration causing 2 coughs. The cough response to the modulation of TRPV-1 by endogenous mediators PGE2 and BK, considered to be indirect sensitizers of the channel, was instead irrespective of the presence of TRPV-1 SNPs. That air pollutants, such as DEP, directly interact with TRPV-1 and cause channel opening (38) suggests that genetics variants also are relevant in the interaction between pollutants and TRPV-1 activation too. This fact, in our opinion, could in part explain epidemiological data which highlight higher COVID-19 mortality in most polluted countries. In summary, TRPV-1 genetic variants and their modulation by air pollutants may play a central role in infection and effects of COVID-19.

## Therapy/Treatment

Based on the above, there is the possibility that TRPV-1 has a relevant role in the infection, susceptibility, and symptoms of COVID-19. This encourages looking at therapeutic agents to down-regulate COVID-19 symptoms and responses TRPV-1-associated, including inflammatory response and cough. Identifying a drug that could down-regulate or inactivate TRPV-1 might therefore provide a protective environment to struggle with SARS-CoV-2 infection and COVID-19 disease.

According to recent data, the inhibition of afferent activity, above all the removal of TRPV-1+ afferent fibers from the lung and airways, could exert a beneficial action on the compromised lung function and clearance of infection (3). Moreover, inactivation of the TRPV-1+ innervation could also lead to better prevention or treatment of ventilator-associated lung injury. Furthermore, several active ingredients of spices including pungent (capsaicinoids) CPS, from red pepper (117), resiniferatoxin, from tropical Euphorbia plants (118), allicin, from onion and garlic (119) and non-pungent (capsinoids) including piperine (black pepper) (120, 121), gingerol and zingerone (ginger) (122), cinnamaldehyde, curcumin (123), eugenol (clove), and camphor, are all TRPV-1 agonists. TRPV-1 is also activated by allyl isothiocyanate (AITC), the organosulfur compound present in horseradish, mustard, and wasabi (124). While the first exposure to TRPV-1 activators may induce acute pain, repeated treatment promotes a refractory state of TRPV-1, named as desensitization. This causes the inhibition of receptor function and stops pain perception, underlying a unique form of analgesia (125). This finding was firstly described for CPS and application of CPS as topical ointments has been applied in clinical use to alleviate chronic painful conditions for decades. The acute desensitization of TRVP-1 occurs within few seconds (~20) after the first administration of vanilloids to the cell. Many signaling pathways including calmodulin, calcineurin, or the decrease of phosphatidylinositol 4,5-bisphosphate, are involved in TRPV-1 desensitization. Oxidative stress reduces phosphatidylinositol 4,5-bisphosphate, and receptor desensitization could be reached at lower doses of agonists in SARS-Cov-2 infection (5). Tachyphylaxis, defined as a reduction in the response after frequent applications of agonists, is another type of desensitization of TRPV-1 by CPS (126).

Patients affected by COVID-19 have been studied in order to evaluate their response to these spices. Consecutive cough-induced challenges were carried out on one of the patients during the recovery phase. The effect of TRPV-1 agonists disappeared in 1–4 h. The duration of this influence increased to around 10 h when small doses of TRPV-1 agonists were added to low-dose broccoli. Paracetamol metabolites, *N*-acetyl-*p*-benzoquinone imine, and *p*-benzoquinone, are TRPV-1 agonists and increased the duration of action of the TRPV-1-broccoli combinations to over 14 h. The results of the challenges suggest a quick short-lasting TRPV-1 desensitization (5, 127). No data until now are available on the treatment of COVID-19 patients with resiniferatoxin (RTX), a known potent agonist of TRPV-1 and active pharmaceutical ingredient that has the potential to be a highly peculiar factor for long-term inactivation of TRPV-1 fibers. Furthermore, COVID-19 patients with mild, moderate, and severe symptoms who received curcumin/piperine treatment promptly recovered from initial symptoms (fever, cough, sore throat, and breathlessness) and exhibited better ability to maintain oxygen saturation above 94% and better clinical outcomes (128). *In silico* drug discovery suggested that curcumin plays as SARS-CoV-2 main protease inhibitor (129). Lastly, some other natural compounds, that are well-known ligands for TRPV-1, may inhibit SARS-CoV-2 as well as lessen some symptoms of COVID-19. Recognized examples are represented by quercetin (130), resveratrol (131), spermidine/spermine (132), naringenin (133), and baicalin. In a prospective, randomized, controlled, and open-label study, a daily dose of 1,000 mg of quercetin was given for 30 days to 152 outpatients affected by COVID-19 to study its adjuvant role in treating the initial symptoms and in preventing the severe effects of the disease. Quercetin was effective in ameliorating COVID-19 early symptoms as well as preventing the severity of the disease (134). Spermidine and spermine, powerful TRPV-1 ligands, have been found to inhibit SARS-CoV-2 infection possibly by promoting viral degradation in the endolysosomes (135). Naringenin, that diminishes TRPV-1 activation channel producing analgesic effect (133), inhibited human coronaviruses infection (136), suggesting that this inhibition can be mediated by TRPV-1. As a final point, baicalin exhibited strong antiviral activities and was recognized as the first non-covalent, non-peptidomimetic inhibitor of SARS-CoV-2 (137). Notably, previous reports showed that baicalin induced down-regulation of TRPV-1 mRNA expression levels in DRG neurons (138). Taken together, evidence gathered from the literature suggests that TRPV-1 can be really considered as target for handling this disease.

## Future Clinical Application

To make our hypothesis clearer and more translational in the clinical setting we envisage future applications that we briefly describe.

### Identify Individuals at Risk of Developing Disease

The analysis of the polymorphic site of the TRPV-1 for deciphering COVID-19 susceptibility could be the key to identify the more vulnerable individuals and those at higher risk for severe disease. As suggested in our previous work (99), the combination of I585, 91S, and 315M modifies the functional properties of the channel and induces an increase in TRPV-1 protein expression due to the multiplied DNA copy number. Furthermore, the corresponding TRPV-1 I585 mutation is associated with a higher risk of wheeze and cough in children with asthma. Since most COVID-19 symptoms are associated with pathways controlled by TRPV-1, we, therefore, expect that the people with 585I, 91S, and 315 M will be more susceptible to adverse effects of COVID-19 infection.

Within the epidemiological area, the identification of TRPV-1 genetic polymorphisms could have important implication in SARS-CoV-2 susceptibility, infection and spread. TRPV-1 genetic variants by increasing the functional properties of the channel could render people more susceptible to virus access into the cell.

### A Tailored Desensitization Treatment

Capsaicin is a common experimental trigger of cough through TRPV-1 activation. However, one-month treatment with oral capsaicin was found to improve cough through a putative desensitization mechanism (139). A recent study (5) reports that administration of a low dose of oral capsaicin (10 and 30 mg of red pepper in capsules) provokes a rapid decrease in induced cough (1–2 min) and nasal obstruction in a single COVID-19 patient, with ultra-rapid clinical effects, suggesting TRPV-1 channel desensitization as the main mechanism. The duration of the effect was around 2 h with 10 mg and 3 h with 30 mg. However, even if the TRPV-1 desensitization does not last long, the repeated treatments (applications) with oral capsaicin or other TRPV-1 agonists (tachyphylaxis) seems able to reduce permanently the symptoms of cough and nasal obstruction that are prevalent in COVID-19 disease.

Along the same lines, a recent publication shows a strong correlation between grams of spice supply pro-capita per day and a decrease in the total number of COVID-19 cases per million population. This suggests that spice consumption, in particular ginger, curcumin, allicin in garlic, which are all TRPV-1 agonists, play a role in fighting COVID-19 (140).

Alternatively, as proposed by Nahama (3) the therapeutic approaches targeting TRPV-1 containing nerve fibers in the lungs, by use of an ultra-potent TRPV1 agonist could modulate the inflammatory and immune signal activity, leading to reduced mortality and better overall outcomes in COVID-19 disease. The potential use of resiniferatoxin, currently in clinical trials for cancer and osteoarthritis pain, as a possible ablating agent of TRPV-1 positive pulmonary pathways in patients with advanced COVID-19 disease, was recommended.

Despite the preliminary evidence and the proposed hypotheses on the therapeutic role of TRPV-1 agonists, future studies are however warranted to test the efficacy and tolerability of these treatments targeting TRPV-1 on patients with COVID-19 disease. Furthermore, research through the use of tailored doses and timing of administration, should confirm these data and mechanisms in order to develop medications, patch tests (capsaicin), nasal sprays, or food supplements based on TRPV-1 desensitization for the treatment of COVID-19 and its main symptoms, including not only cough but also pain and tachycardia. These studies should be corroborated by the genetic characterization of patients with COVID-19 by six nonsynonymous functional polymorphisms of TRPV-1 (K2N rs9894618, P91S rs222749, I315 M rs222747, T469I rs224534, T505A rs17633288, and I585V rs8065080), that determine a substantial difference in capsaicin sensitivity with levels of SNP-based responsiveness ranging from 2 to 6. Based on our previous study we hypothesized that the most responsive individuals will need a lower dose of agonist (capsaicin) to induce the same effect than fairly ones. This would help to design tailored strategies for SARS-CoV-2 infection too.

## Conclusion

The battle against the new coronavirus will be still long, so know the mechanisms of TRPV-1, a receptor involved in lung defense mechanisms, inflammation, and immunomodulation might be relevant in the susceptibility to SARS-CoV-2 infection. Novel target polymorphic TRPV-1 receptor could be the key factor in COVID-19 susceptibility to design not only preventive but also therapeutic strategies in SARS-CoV-2 infections. Exploring the role of multiple SNPs of the TRPV-1 gene in the sensitivity to lung and heart dysfunction in SARS-CoV-2 infection will open new therapeutic approaches targeting TRPV-1 that could modulate the inflammatory and immune signal activity leading to a better overall outcome.

## Author Contributions

SP and FL conceived the manuscript. SP, FL, PM, and MC were involved in early discussions and mapping the concepts that led to this paper and wrote the first draft of the manuscript. All authors read and critically reviewed drafts of the manuscript.

## Funding

This study was supported by the BIRD175721 funding, provided by the University of Padova, Department of Cardio-Vascular-Thoracic Science and Public Health.

## Conflict of Interest

The authors declare that the research was conducted in the absence of any commercial or financial relationships that could be construed as a potential conflict of interest.

## Publisher's Note

All claims expressed in this article are solely those of the authors and do not necessarily represent those of their affiliated organizations, or those of the publisher, the editors and the reviewers. Any product that may be evaluated in this article, or claim that may be made by its manufacturer, is not guaranteed or endorsed by the publisher.

## Abbreviations

IL-6: Interleukin 6
IL-2: Interleukin 2
IL-7: Interleukin 7
IL-10: Interleukin 10
GSCF: Granulocyte colony-stimulating factor
IP-10: Interferon ɤ-induced protein
MCP1: Monocyte chemoattractant protein-1
MIP1A: Macrophage inflammatory protein-1α
TNFα: Tumor Necrosis Factor α
TRPV-1: Transient receptor potential vanilloid subtype 1
CPS: Capsaicin
DEP: Diesel exhaust particulate
ECG: Electrocardiogram
HRV: Heart rate variability
PGE2: Prostaglandin E2
BK: Bradykin
HRV: Human rhinovirus
RSV: Respiratory syncytial virus
MV: Measles virus
HCV: Hepatitis C virus
HSV-2: Herpes simplex virus type 2
HSV-1: Herpes simplex virus type 1
VZV: Varicella-zoster virus.
